# Macromolecular organization and fine structure of the human basilar membrane - RELEVANCE for cochlear implantation

**DOI:** 10.1007/s00441-014-2098-z

**Published:** 2015-02-07

**Authors:** Wei Liu, Francesca Atturo, Robair Aldaya, Peter Santi, Sebahattin Cureoglu, Sabrina Obwegeser, Rudolf Glueckert, Kristian Pfaller, Annelies Schrott-Fischer, Helge Rask-Andersen

**Affiliations:** 1 Department of Surgical Sciences, Head and Neck Surgery, section of Otolaryngology, Uppsala University Hospital, 751 85 Uppsala, Sweden; 2 Department of Neurology, Mental Health and Sensory Organs Otorhinolaryngologic Unit,, Medicine and Psychology Sapienza, Rome, Sweden; 3 Department of Neurology, Mental Health and Sensory Organs, Otorhinolaryngologic Unit, Medicine and Psychology, Sapienza, Rome; 4 Department of Surgical Sciences, Section of Otolaryngology Uppsala University Hospital, SE 751 85 Uppsala, Sweden; 5 University Hospital Innsbruck-TILAK, University Hospital Innsbruck-Tirol Kliniken, Anichstrasse 35, 6020 Innsbruck, Austria; 6 Department of Otolaryngology, Medical University of Innsbruck, Anichstrasse 35, 6020 Innsbruck, Austria; 7 University Hospital Innsbruck-Tirol Kliniken, Anichstrasse 35, 6020 Innsbruck, Austria; 8 Department of Otolaryngology, University of Minnesota, 121, Lions Research Bldg., 2001 Sixth St. SE, Minneapolis, MN 55455 USA; 9 Department of Histology and Molecular Cell Biology, Institute of Anatomy and Histology, Medical University of Innsbruck, Innsbruck, Austria

**Keywords:** Basilar membrane, Human, Collagen II, Ultrastructure, Cochlear implant

## Abstract

**Introduction:**

Cochlear micromechanics and frequency tuning depend on the macromolecular organization of the basilar membrane (BM), which is still unclear in man. Novel techniques in cochlear implantation (CI) motivate further analyses of the BM.

**Materials and methods:**

Normal cochleae from patients undergoing removal of life-threatening petro-clival meningioma and an autopsy specimen from a normal human were used. Laser-confocal microscopy, high resolution scanning (SEM) and transmission electron microscopy (TEM) were carried out in combination. In addition, one human temporal bone was decellularized and investigated by SEM.

**Results:**

The human BM consisted in four separate layers: (1) epithelial basement membrane positive for laminin-β2 and collagen IV, (2) BM “proper” composed of radial fibers expressing collagen II and XI, (3) layer of collagen IV and (4) tympanic covering layer (TCL) expressing collagen IV, fibronectin and integrin. BM thickness varied both radially and longitudinally (mean 0.55–1.16 μm). BM was thinnest near the OHC region and laterally.

**Conclusions:**

There are several important similarities and differences between the morphology of the BM in humans and animals. Unlike in animals, it does not contain a distinct pars tecta (arcuate) and pectinata. Its width increases and thickness decreases as it travels apically in the cochlea. Findings show that the human BM is thinnest and probably most vibration-sensitive at the outer pillar feet/Deiter cells at the OHCs. The inner pillar and IHCs seem situated on a fairly rigid part of the BM. The gradient design of the BM suggests that its vulnerability increases apical wards when performing hearing preservation CI surgery.

**Electronic supplementary material:**

The online version of this article (doi:10.1007/s00441-014-2098-z) contains supplementary material, which is available to authorized users.

## Introduction

**Fig. 1:**
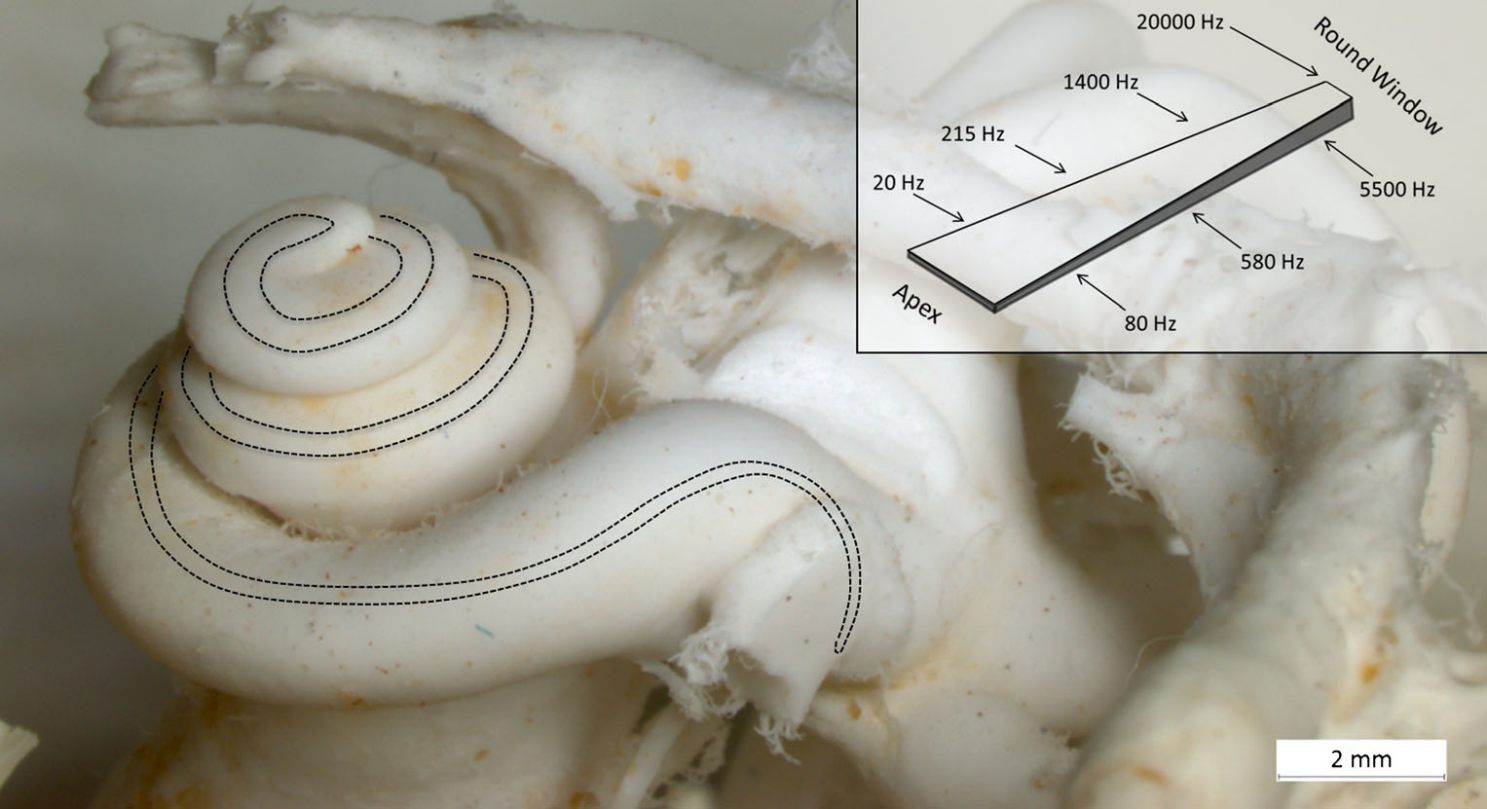
Plastic mould of a left human cochlea with BM projected and drawn to scale. BM width increases successively from the round window to the apex while its thickness decreases (*inset*)

The human cochlea contains approximately 15 000 sensory hair cells located in an epithelial ridge anchored on a micro-thin, 34-mm-long spiral membrane called the basilar membrane (BM) (Fig. 1). The BM has undergone spectacular evolution and adaptation in various mammals (Vater 1988). Sound-evoked vibrations are transmitted into the ear and produce frequency/place-dependent BM motions in the cochlea. These motions are converted into an electric code in the auditory nerve by the sensory cells (Helmholtz 1863; Wever et al. 1954; Zwislocki 1948; Békésy 1960). Micromechanics depend on the gradient stiffness and coupling between adjacent structures in the BM. However, its macromolecular organization in man is still unclear. Studies of BM structure and composition have been performed in animal models (Iurato 1958; Angelborg and Engström 1974; Cabezudo 1978; Katori et al. 1993; Mikuni et al. 1994, 1995), but there are few studies in man (Corti 1851; Retzius 1884; Ishiyama et al. 2009; Yamashita et al. 1991). Here, we analyze the configuration of the human BM. For this purpose, we use laser confocal immunohistochemistry, transmission (TEM) and scanning electron microscopy (SEM) of freshly fixed, non-pathological (mostly normal hearing; see Table 1) human cochleae obtained at surgery. These specimens can occasionally be harvested with immediate fixation in the operating room to preserve anatomy and molecular structure. In addition, a decellularized human cochlea was analyzed.

**Table 1:** Data of the patients and application of the cochleae

Age (years)	Gender	PTT	Analysis
Middle-aged	Female	50 dB (1kH to 8kH)	IHC (surgical, meningioma)
Middle-aged	Male	Normal	IHC (surgical, meningioma)
>70	Male	50 dB (2kH to 4kH)	IHC (surgical, meningioma)
Middle-aged	Female	Normal	TEM (surgical, meningioma)
Middle-aged	Male	Normal	TEM (surgical, meningioma)
Middle-aged	Female	Normal	SEM (surgical, meningioma)
Middle-aged	Female	Normal	SEM (surgical, meningioma)
67	Female	Normal	sTSLIM (autopsy, meningioma)
57	Female	Normal	TEM (peril.perf.)
57	Male	Normal	TEM (peril.perf.)
Middle-aged	Male	Deaf	TEM (surgical, m. schwannoma)

All patients had subjectively normal hearing

*PTT* pure tone threshold, *TEM* transmission electron microscope, *SEM* scanning electron microscope, *sTSLIM* scanning thin-sheet laser illumination microscopy, *IHC* immunohistochemistry, *peril. Perf.* perilymphatic perfusion in Innsbruck, *surgical* cochlear specimens obtained at surgery in Uppsala, *m. schwannoma* malignant schwannoma

We found that the human BM consists in acellular, highly organized extracellular matrix (ECM) layers mainly composed of laminin-β2 and collagen types IV and II. Beneath is a cellular layer named the tympanic covering layer (TCL) containing blood vessels (also named tympanic mesothelial cells by Bhatt et al. 2001). The layers organization changed along the spiral length of the BM. The relevance of this anisotropy for cochlear implantation is discussed.

## Material and methods

Studies on human materials were approved by the local ethics committee (no. 99,398, 22/9 1999, cont., 2003 and Dno. 2013/190) and patient consent was obtained. Studies of animal cochlea were approved by the local ethics committee (nos. C254/4, C209/10). Studies adhered to the rules of the Helsinki declaration.

### SEM

Two cochleae were used that had been previously processed and morphologically analyzed (Rask-Andersen et al. 2000; Table 1). Maximal resolution at this voltage was estimated to approx. 2 nm. Digital photos were taken at 1,280–1,024 ppi resolution. Measurements were performed using image analysis software Image Pro 4.5.1.29 (Media Cybernetics, MD, USA). BM widths were calculated from the tympanic lip to the basilar crest. The ratio between BM and scala tympani width assessed parallel was also derived. BM thickness was evaluated at three different locations: near the spiral ligament insertion (SL), under the outer pillar foot (OPF) and at spiral lamina insertion (OSL). The TCL thickness was estimated beneath inner and outer hair cells. Measurements were made at different cochlear turns. The decellularized human temporal bone was critical point dried with carbon dioxide, coated with gold-palladium in a sputter coater and imaged using a Hitachi S3500N Variable Pressure Scanning Electron Microscope at the University of Minneapolis.

### TEM

Earlier specimens collected both at surgery and after perilymphatic perfusion were analyzed at the ear research laboratories in Uppsala (*n* = 3) (Rask-Andersen et al. 2000; Tylstedt et al. 1997) and Innsbruck (*n* = 2). The specimens used for TEM had been fixed in 3 % phosphate-buffered glutaraldehyde and rinsed in cacodylate buffer, followed by fixation with 1 % osmium tetraoxide at 4 °C for 4 h. The specimens were infiltrated with Epon in a vacuum chamber for 4 h. For TEM analysis, sections were viewed in a JEOL 100 SX electron microscope (Uppsala) and in Zeiss LIBRA (Institute of Zoology, Innsbruck) and Philips CM 120 (Division of Anatomy, Histology and Embryology, Innsbruck) transmission electron microscopes.

### Decellularization

A fresh autopsy human temporal bone specimen was removed from a normal human and decellularized with a detergent (sodium dodecyl sulfate) as previously described (Santi and Johnson, 2013). This specimen was prepared for imaging by scanning thin-sheet laser illumination microscopy (sTSLIM) and then rehydrated, fixed with osmium tetroxide and critical point dried for SEM.

### Fixation and sectioning of human cochlea for immunohistochemistry

Three cochleae from three adult patients (Table 1), with normal hearing (*n* = 1) or hearing loss limited to certain high frequencies (*n* = 2), were dissected out as a whole piece during petro-clival meningioma surgery. In the operating room, the cochleae were immediately placed in 4 % paraformaldehyde diluted with 0.1 M phosphate buffered saline (PBS; pH 7.4). After a 24-h fixation, the fixative was replaced with 0.1 M PBS then with 10 % EDTA solution at pH 7.2 for decalcification. After 4 weeks, the thoroughly decalcified cochleae were rinsed with PBS. For frozen sections, the cochleae were embedded in Tissue-Tek (OCT Polysciences), rapidly frozen and sectioned at 8–10 μm using a Leica cryostat microtome. The frozen sections were collected onto gelatin/chrome-alum-coated slides and stored below −70 °C before immunohistochemistry (Liu et al. 2011, 2012).

### Antibody and immunohistochemistry

The antibody against laminin-β2 was a monoclonal antibody from rat (catalog number # 05–206, dilution 1:100; Millipore, Billerica, MA, USA). It recognizes and is specific for the β2 chain laminin. It does not cross-react with other basement membrane components or fibronectin (FN). The type IV collagen antibody was a goat polyclonal antibody (catalog number AB769, dilution 1:10; Millipore). It has less than 10 % cross-reactivity with collagen types I, II, III, IV, V and VI. The type II collagen antibody was a mouse monoclonal antibody (catalog number CP18, dilution 1:100; Millipore).

The antibody against neuron-specific class III beta-tubulin (Tuj1) was a polyclonal antibody (catalog number # 04–1,049, dilution 1: 200; Millipore). Another tubulin antibody was a monoclonal antibody from mouse (catalog MAB1637, dilution 1: 200; Millipore). Elastin antibody was a mouse monoclonal Ab (MAB2503; Millipore). Fibronectin antibody was a mouse monoclonal antibody (MAB88916 or Roche 1,087,720; Millipore) reacting with human fibronectin cell attachment fragment. It inhibits the attachment of normal rat kidney cells to human fibronectin-coated plastic surface. The antibody for Integrin beta1 (sc-9,970; Santa Cruz Biotechnology) was a mouse monoclonal antibody against full length integrin B1 of human origin. The anti-collagen IX antibody (ab134568; Abcam) was a rabbit polyclonal antibody to collagen IX. Its specificity was tested using western blotting. In our control experiment using human skin, the epithelial cells were very well labeled. Antibody combination, characteristics and sources are summarized in Table 2. Immunohistochemical (IHC) procedures on cochlear sections have been described in previous publications (Liu et al. 2011, 2012). Briefly, incubation of sections on slides with solution of the antibodies was carried out under humid atmosphere at 4 °C for 20 h. After rinsing with PBS (3 × 5 min), the sections were subsequently incubated with secondary antibodies conjugated to Alexa Fluor 488 and 555 (Molecular Probes, Carlsbad, CA, USA), counter-stained with a nuclear stain DAPI (4′,6-diamidino-2-phenylindole dihydrochloride) for 5 min, rinsed with PBS (3 × 5 min) and mounted with a VECTA SHIELD (Vector Laboratories, Burlingame, CA, USA) mounting medium. The sections used for antibody control were incubated with 2 % bovine serum albumin (BSA) omitting the primary antibodies. As a result of the control experiment, there was no visible staining in any structure of the cochleae.

**Table 2:** Antibodies used

Antibody	Dilution	Type	Host	Clone	Catalog no.	Producer
Laminin-β2	1:100	Mono	Rat	A5	05-206	Millipore
Type IV collagen	1:10	Poly	Goat	N/A	AB769	Millipore
Type II collagen	1:50	Mono	Mouse	N/A	CP18	Millipore
Type IX collagen	1:50	Poly	Rabbit	N/A	AB134568	Abcam
Type XI collagen	1:100	Poly	Rabbit	N/A	AB64883	Abcam
Elastin	1:50	Mono	Mouse	N/A	MAB2503	Millipore
Fibronectin	1:50	Mono	Mouse	N/A	MAB88916	Millipore
Integrin β1	1:50	Mono	Mouse	N/A	sc-9,970	Santa Cruz
Tuj1	1:200	Mono	Rabbit	N/A	04-1,049	Millipore
Tuj1	1:200	Mono	Mouse	N/A	MAB1637	Millipore
Emilin 2	1:50	Poly	Rabbit	N/A	HPA040739	Sigma
Tenascin	1:50	Poly	Rabbit	N/A	sc-20,932	Santa Cruz

*Poly* polyclonal antibody, *mono* monoclonal antibody

### Imaging and photography

Stained sections were investigated with an inverted fluorescence microscope (Nikon TE2000; Nikon, Japan) equipped with a spot digital camera with three filters (for emission spectra maxima at 358, 461 and 555 nm). Both microscope and camera are connected to a computer system installed with image software (NIS Element BR-3.2; Nikon) including image merging and a fluorescence intensity analyzer. For laser confocal microscopy, the same microscope was used, which is equipped with a laser emission and detection system with three different channels. The optical scanning and image-processing tasks were run by the program Nikon EZ-C1 (v.3.80) including reconstruction of Z-stack images into projections or 3D images.

## Results

The BM showed structural anisotropy along its length. Dimensions, gross morphology and macromolecular organization changed from base to apex as well as radially. According to earlier reports, the width of the human BM can be divided into two separate regions (Bhatt et al. 2001). The pars pectinata is defined as the layer between the foot of the outer pillar cell and the basilar crest of the spiral ligament. The pars tectum is defined as the layer between the outer pillar cell and the tympanic lip of the spiral limbus. It corresponds to the parts described in animals as pars pectinata and arcuata, respectively. In our human specimens, a similarly clear morphologic separation could not be distinguished, except in the hook region where the BM thickness increased lateral to the outer pillar feet.

### SEM

**Fig. 2:**
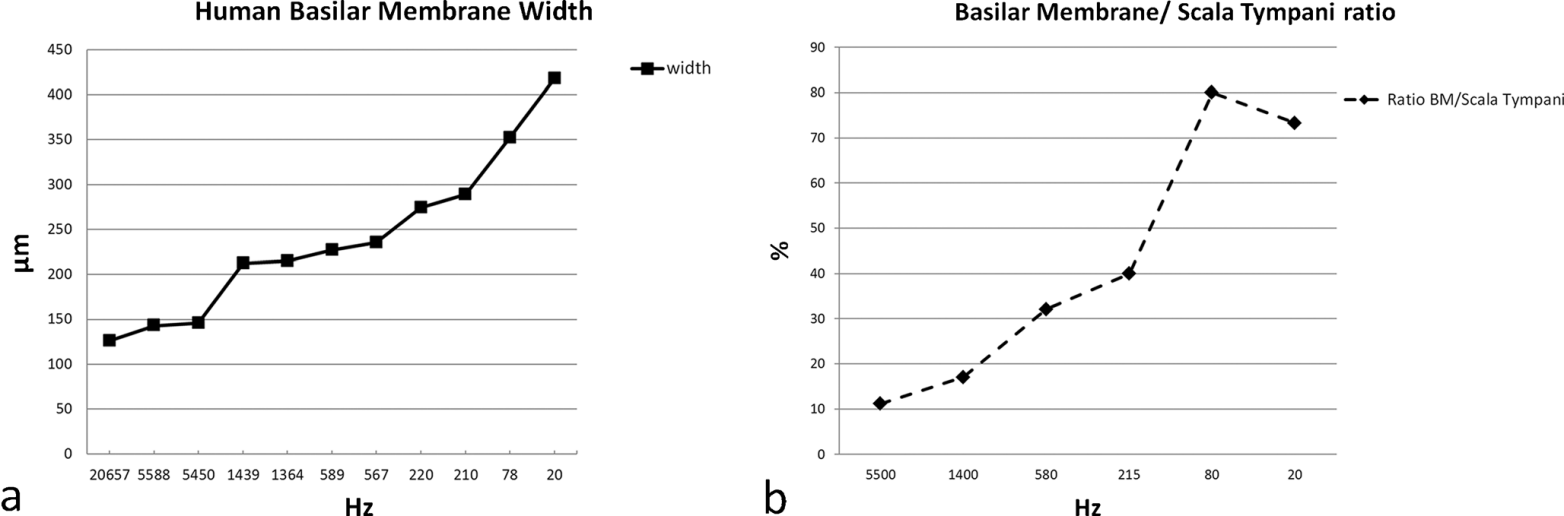
BM width (**a**) and the ratio of BM/scala tympani widths (**b**) shown in Table 2

**Fig. 3:**
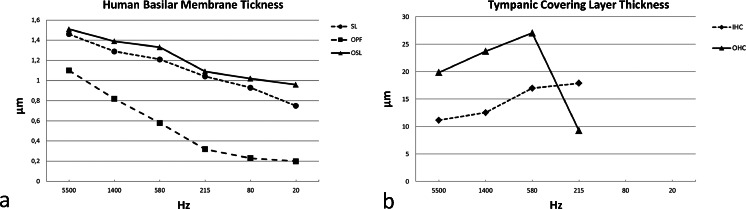
BM (**a**) and TCL (**b**) thicknesses at different frequency locations assessed from one radial sectioned SEM specimen of a normal hearing individual, mid-age female. BM dimensions were estimated at spiral ligament (*SL*), outer pillar foot (*OPF*) and spiral lamina insertion (*OSL*). TCL thickness was estimated beneath inner (*IHC*) and outer hair cells (*OHC*)

Thickness and width were calculated from a mid-modiolar, hemi-sectioned SEM specimen from a normal hearing mid-aged female. Figure 1 demonstrates the BM that is projected and drawn to scale in a plastic mould of a human cochlea. The BM width increased successively from the round window to the apex while its thickness decreased (Figs. 2a, 3a; Tables 3, 4). The width of the BM ranged from 126 μm at the base to 418 μm in the apex (Figs. 2a, 3a). The BM/scala tympani relative width (measured in parallel) ranged from 11.2 % in the base to 80.0 % in the apex (Table 4; Fig. 2b). Mean thickness of BM measured at the SL, OPF and OSL decreased almost linearly towards the apex. Measured with SEM, BM was thinnest beneath the outer pillar feet (OPF/OHCs) and Claudius cells (Table 3, Fig. 3a). The thickness at the OPF varied between 1.10 μm at the lower basal turn (approx. 5,500 Hz) to 0.20 μm at the apex. TCL thickness increased apically (Fig. 3b). In the apical region, TCL thickness was drastically reduced beneath the OHCs.

**Table 3:** Human BM and TCL thicknesses relative location and estimated frequency region according to the Greenwood place/frequency scale (estimated values from a radial sectioned SEM specimen of a normal hearing individual, mid-age female)

Anatomical cochlear region (turn)	Frequencies (Hz)	BM thickness (μm)			TCL thickness (μm)	
		SL	OPF	OSL	IHC	OHC
Lower basal	5,500	1.46	1.10	1.51	11.14	19.84
Upper basal	1,400	1.29	0.82	1.39	12.52	23.70
Lower middle	580	1.21	0.58	1.33	16.97	27.04
Upper middle	215	1.04	0.32	1.09	17.87	9.24
Lower apical	80	0.93	0.23	1.02	–	–
Apex	20	0.75	0.20	0.96	–	–

*BM* Basilar membrane, *TCL* tympanic covering layer, *SL* spiral ligament, *OPF* outer pillar feet, *OSL* osseous spiral lamina, *IHC* inner hair cell, *OHC* outer hair cell

**Table 4:** Human BM and scala tympani width relative position and estimated frequency region according to the Greenwood place/frequency scale (estimated values from a radial sectioned SEM specimen of a normal hearing individual, mid-age female)

Anatomical cochlear region (turn)	Frequencies (Hz)	BM width (μm)	ST width (mm)	BM/ST ratio (%)
Lower basal	5,500	144.5	1.29	11.2
Upper basal	1,400	213.5	1.25	17.0
Lower middle	580	231	0.72	32.0
Upper middle	215	281.5	0.70	40.1
Lower apical	80	352	0.44	80.0
Apex	20	418	0.57	73.3

*BM* Basilar membrane, *ST* scala tympani

**Fig. 4:**
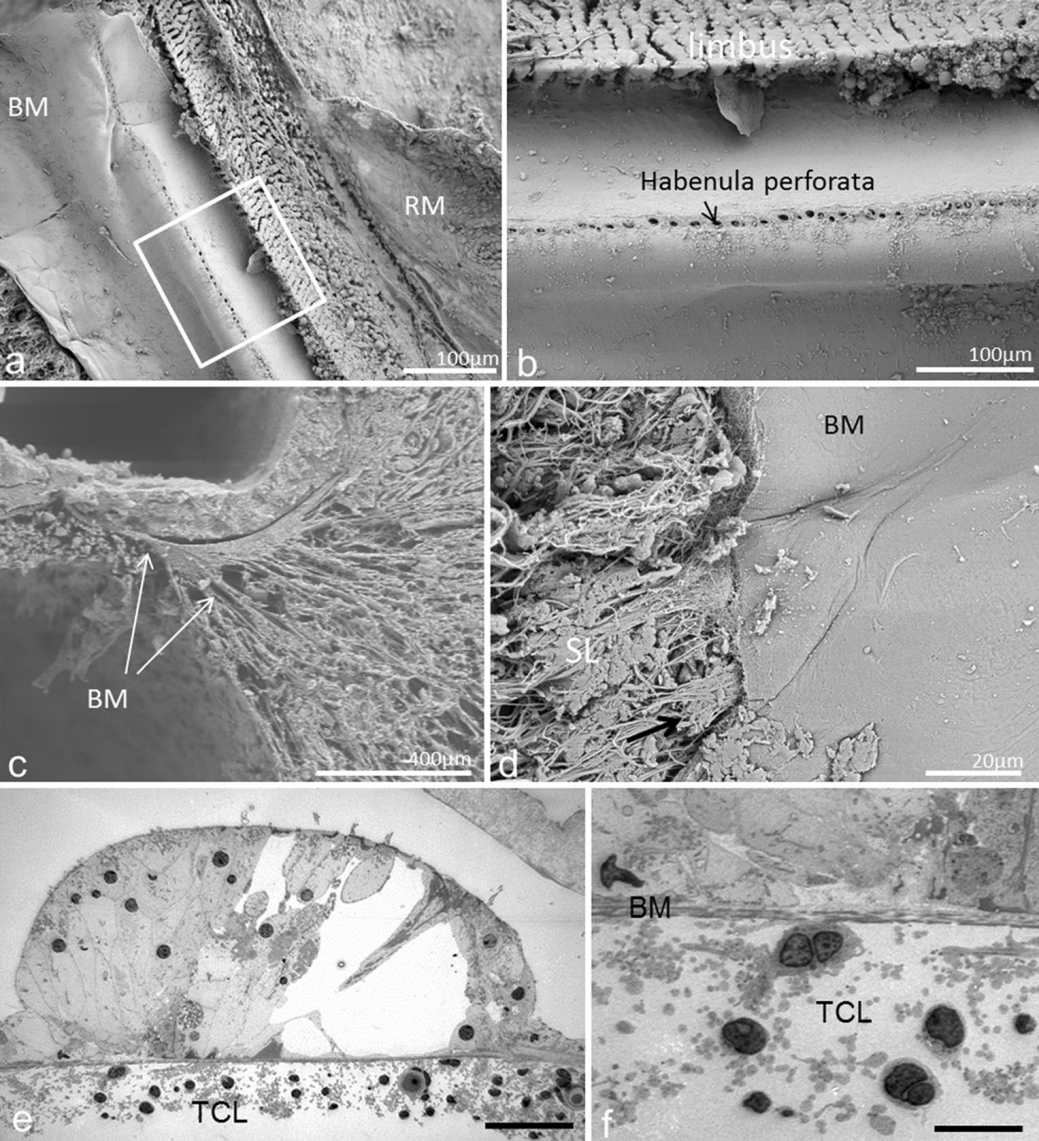
SEM and TEM of the human BM. **a** Limbus dentate cells and BM viewed from endolymph side. **b** Framed area in (**a**) shown in higher magnification. The limbus dentate cells are seen (upper aspect) as well as the habenula perforata. **c** BM attachment to the spiral ligament at basilar crest (*arrows*). **d** Surface view shows anchoring of BM to the spiral ligament (*SL*). **e** Low-power TEM of human organ of Corti at the low-frequency region and the tympanic cover layer (*TCL*). **f** Higher-power TEM of the BM including the TCL

**Fig. 5:**
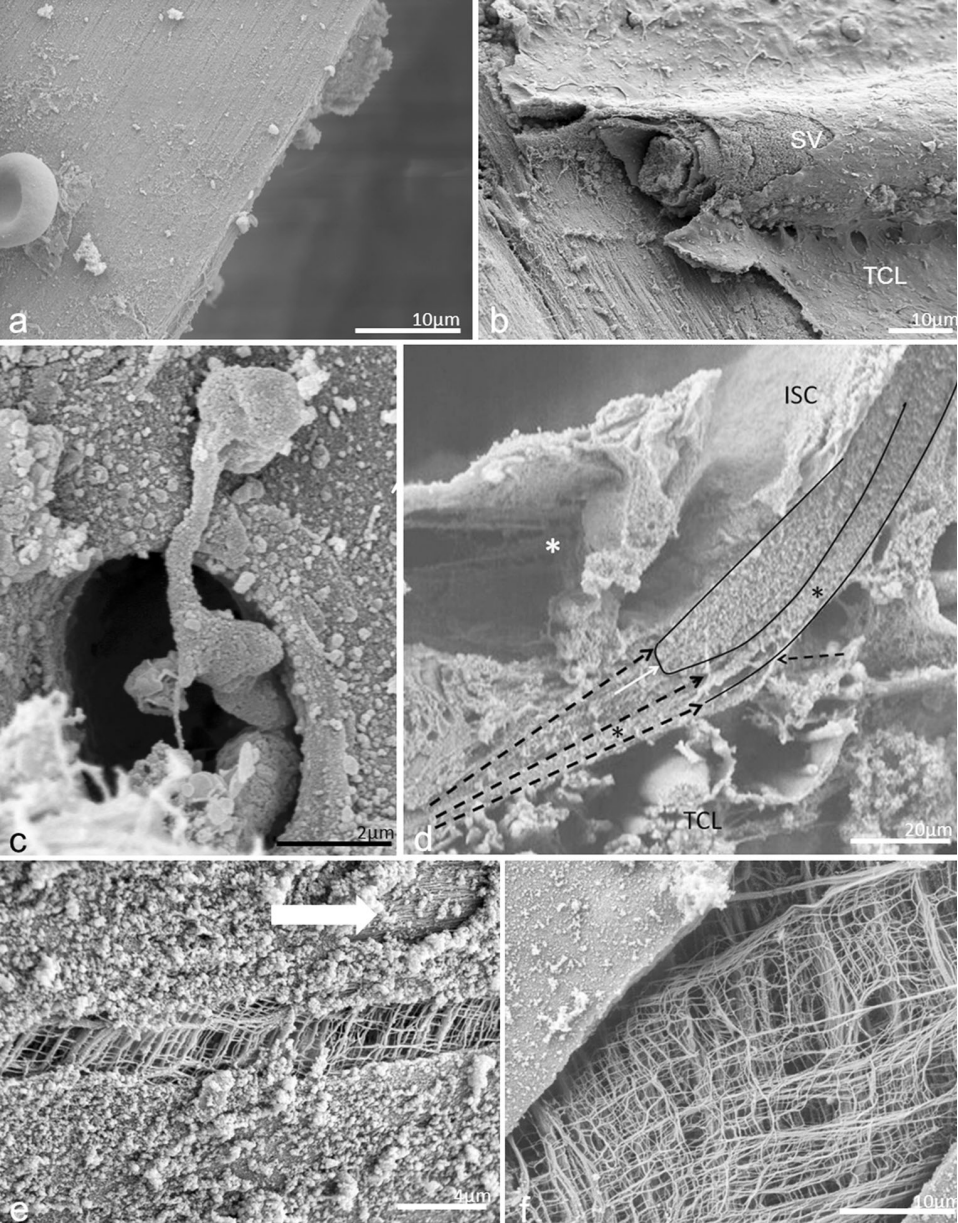
SEM of human BM. **a** BM of the basal turn projected from the scala media. **b** BM from the lower basal turn; *SV* spiral vessel, *TCL* tympanic covering layer. **c** SEM of a “habenula opening” with afferent nerve terminal. **d** SEM of radially sectioned BM at the tympanic lip. The BM thickens as it merges with the tympanic lip. A leaflet continues beneath the tympanic lip (*). **e** BM viewed from scala media. Radial fibers are exposed at *arrow*. **f** BM viewed from scala media. The basement membrane is ruptured and collagen fibers are exposed

In one SEM specimen, the organ of Corti was accidently removed and the epithelial basement membrane was exposed as a “carpet-like” structure (Fig. 4a–d). The scala media view revealed a series of neural openings (habenula perforate) into the sensory epithelium (Fig. 4a, b). Beneath the basement membrane was a fibrous layer extending from the tympanic lip of the lamina spiralis to the basilar crest of the spiral ligament. It represented the “proper BM” and consisted of various sized radial parallel fiber bundles (10–20 nm in diameter) (Fig. 5a, b, e, f). Lateral to the basilar crest, in the spiral ligament, the BM thickened and formed an “anchor-like” structure from which several fibers emerged (Fig. 4c, d). The BM also displayed longitudinally arranged fibrils (Fig. 5e, f). The BM merged with the tympanic lip. At this point, it also widened and formed two in-distinct layers (Fig. 5d). Both layers continued in the spiral lamina of the tympanic lip.

### TEM

The detailed structure of the BM was best visualized with TEM. The BM identified with SEM seemed to correspond to the electron-dense fibrous layer observed with TEM. In the basal turn, in radial sections, this layer was mostly homogenously stained while in tangential sections the fibers appeared like “strings”.

**Fig. 6:**
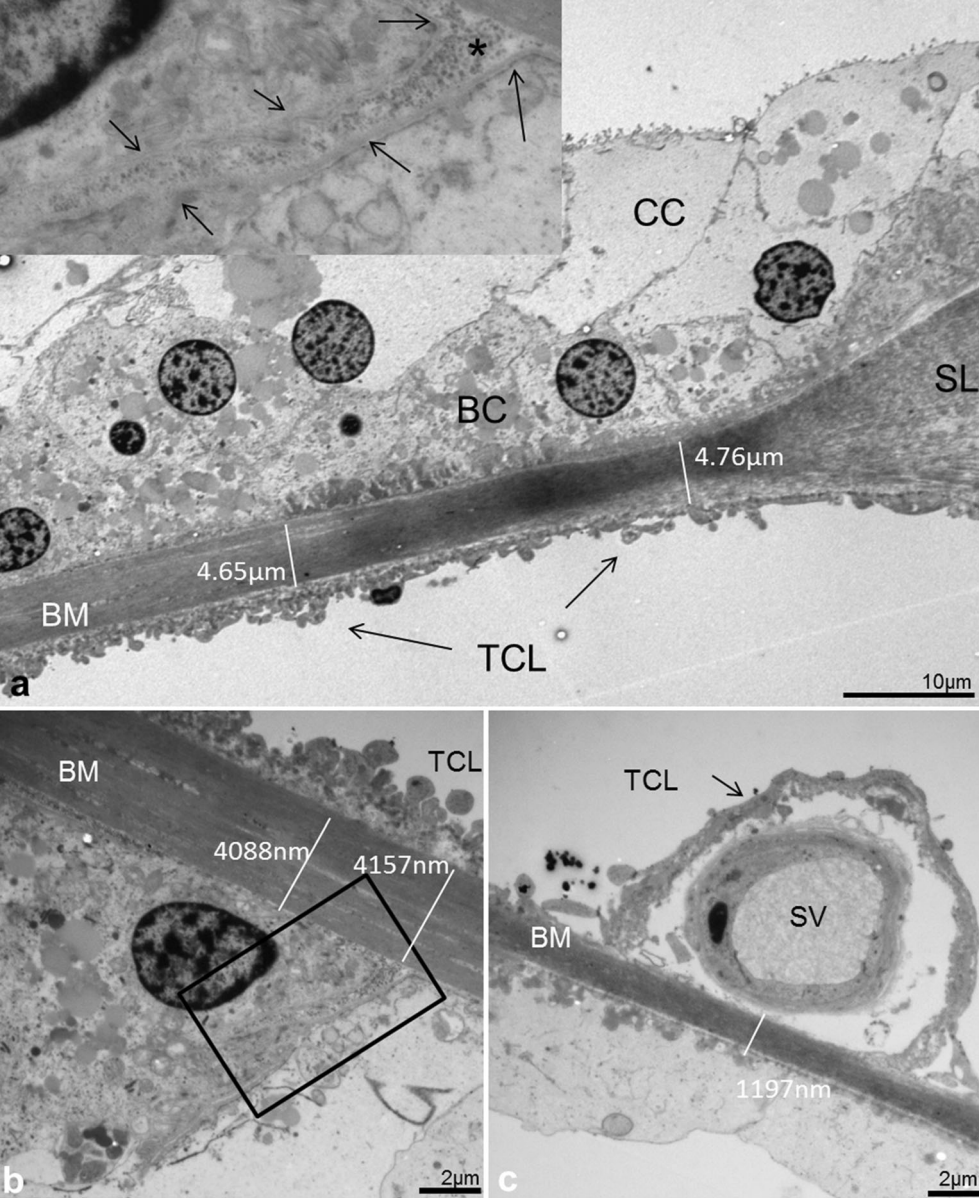
Thickness variation of the human BM in the hook (high-frequency) region portrayed with TEM. **a** Radial section shows the alternate radial thickness of the BM at the “hook” region. Laterally, the organ of Corti contains both Claudius (*CC*) and Boettcher cells (*BC*). The BM is over 4 μm thick and widens at the spiral ligament (*SL*). The electron-density of the BM is partly reduced and gives the impression that the BM is thinner near the SL. The tympanic covering layer (*TCL*) is thin. **b** BM thickness is slightly reduced medially beneath a Boettcher cell. An invagination of the basement membrane is seen between a Boettcher and Hensen cell (*arrows*, *framed area* is magnified in *inset* in **a**). **c** BM thickness is additionally reduced medially beneath the pillar feet at the spiral vessel (*SV*)

In the hook region, this layer was thicker and homogeneously stained except at the basilar crest where the electron-density of the BM was partly reduced giving the “illusion” that the BM narrowed (Fig. 6). Beneath the Claudius and Boettcher cells, the BM was almost 5 μm thick. Medially, beneath the spiral vessel and inner pillar cell foot, the BM thickness diminished to approximately 1.2 μm. Invaginations of the basement membrane were seen between Boettcher and Hensen cells (Fig. 6a, inset in b).

**Fig. 7:**
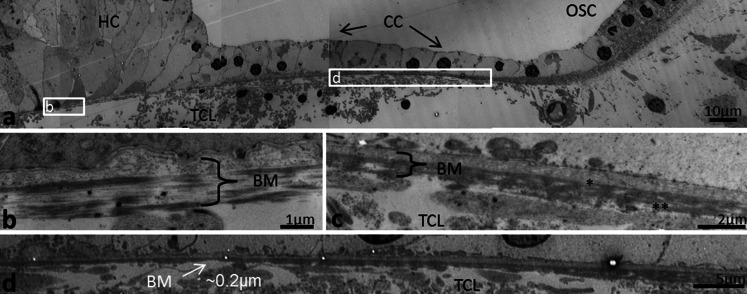
Radial thickness of the collagen II layer in the low-frequency region portrayed with TEM. **a** Radial section shows the alternate radial thickness of the BM at the second turn. Laterally, the organ of Corti contains Claudius cells (*CC*). Contrary to the high-frequency region, the radial thickness is reduced laterally. **b** BM thickness beneath Hensen cells (*HC*) is around 1 μm. Collagen II layer is less homogeneous and the basement membrane is interrupted and multi-layered. **c** Collagen II layer (**) and basement membrane (*) beneath Claudius cells (*CC*). **d** *Framed area* in (**a**) shown in higher magnification. Collagen II layer is extremely thin (*arrow*). *TCL* tympanic covering layer, *OSC* outer sulcus cells

In the second turn, BM was thinner and less homogeneously stained (Fig. 7). Basement membrane invaginations with extra-cellular tissue penetrating between Claudius and Hensen cells were found. In addition, the basement membrane was often interrupted and multi-layered (Fig. 7b, c). The fibrous layer was thinnest beneath the Claudius cells, where the electron-dense layer sometimes appeared to be non-existent in radial sections (Fig. 7d). Thus, TEM measurements showed that the gradient radial thickness of the BM in the low- and high-frequency regions was inverted. That is, in the hook region, the radial thickness increased laterally while in the second turn it decreased. The minimum thickness of the BM (proper) fibrous layer at the Claudius cells differed more than 20 times (0.2–4.8 μm) between the high- and low-frequency regions (hook and second turn).

### Tympanic covering layer (TCL) – SEM, TEM

**Fig. 8:**
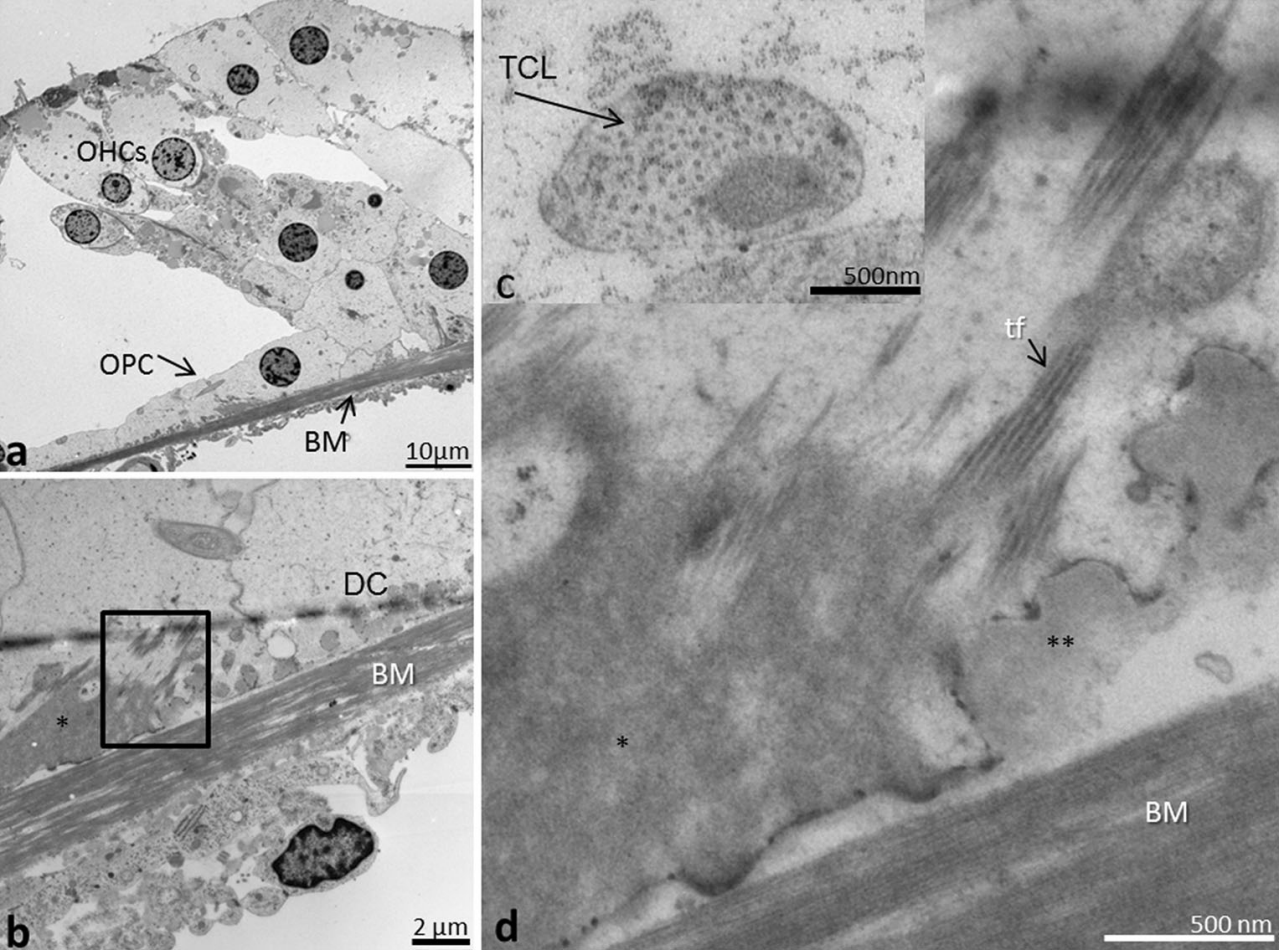
TEM of human BM, high-frequency region. **a** Low-power view of outer hair cells region (*OHC*) in the lower basal turn. **b** Medium-power view of Deiter cells (*DC*) showing the filament cone and BM. *Framed area* is magnified in (**d**). **c** TEM of a cell process of the TCL containing a rich amount of 25 Å micro-tubuli. **d** *Framed area* in (**b**) shown in higher magnification. The basal part of the Deiter cell contains a tonofilament cone (*tf*). The filaments are anchored in a more electron-dense cytoplasmic region of the cell that probably contains actin (*). **Exocytotic vesicles

**Fig. 9:**
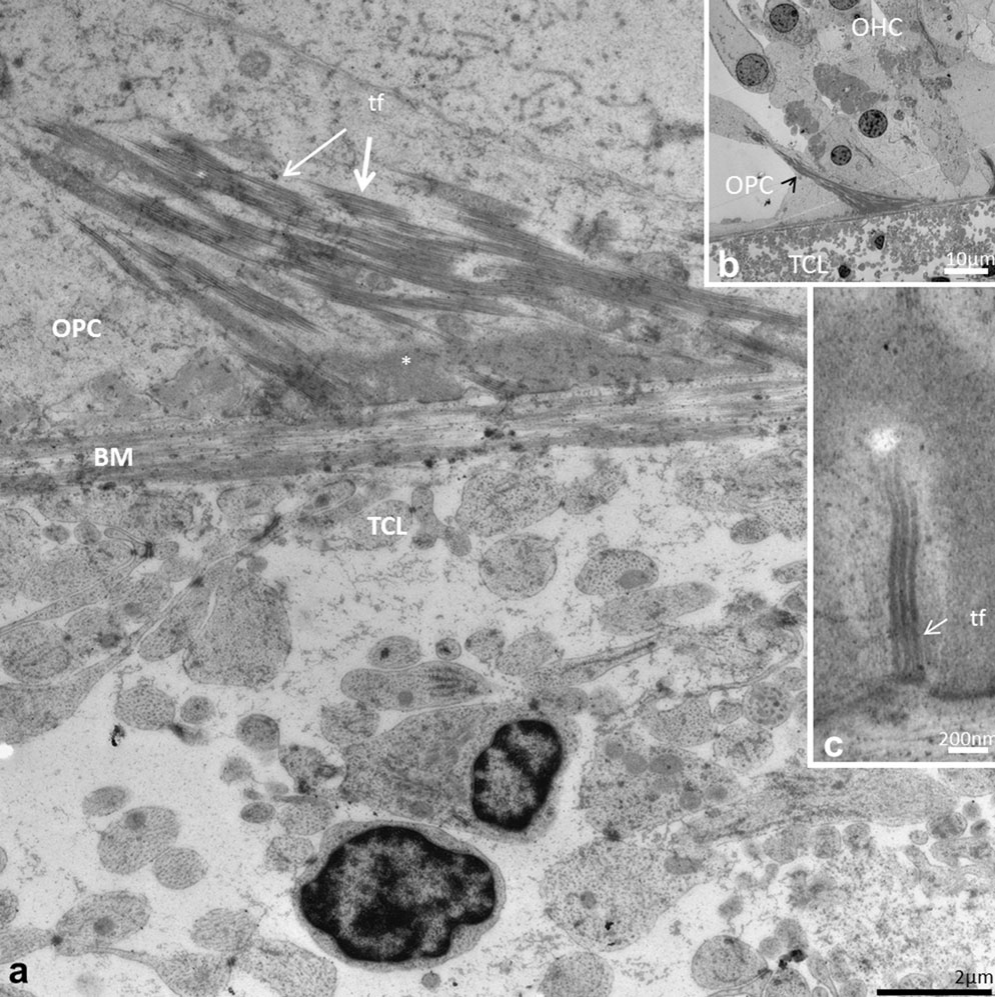
TEM of human organ of Corti and BM in the low-frequency region. **a** Tonofilaments in the outer pillar cell (OPC) and BM at the upper turn of the cochlea. Outer hair cell (OHC) region is shown in low magnification in the *upper inset* (**b**). *Lower inset* (**c**) shows tonofilament anchoring in cytoplasmic, electron-dense area and in plasmalemmal densities facing the BM. *tf* tonofilament, * cytoplasmic density

The TCL contained cells and blood vessels in an extra-cellular ground substance (Fig. 6, 7, 8, 9). TCL thickened apically (Figs. 3b, 13a, see below) but was thinner beneath the OHCs in the apex. In the lower basal turn, the TCL was thinner and contained flat mesenchymal cells with short and long processes (Fig. 16a). These cell processes contained microtubules (Fig. 8c). The TCL continued into the wall of the spiral ligament of the lateral surface wall of the scala tympani. The TCL cell processes often enclosed the spiral vessels (Figs. 6b, 14a, see below).

### Interaction between BM and sensory epithelium - TEM

Inner and outer pillar and Deiter cells exhibited electron-dense intermediate cytoskeleton filaments representing tonofilaments. These filaments ended in focal densities of the basal plasmalemma (Fig. 8) near the BM (Figs. 8, 9). A rich number of exocytotic vesicles were seen nearby (Fig. 8d), often with a structurally modified basement membrane (Fig. 8d).

### Decellularization

**Fig. 10:**
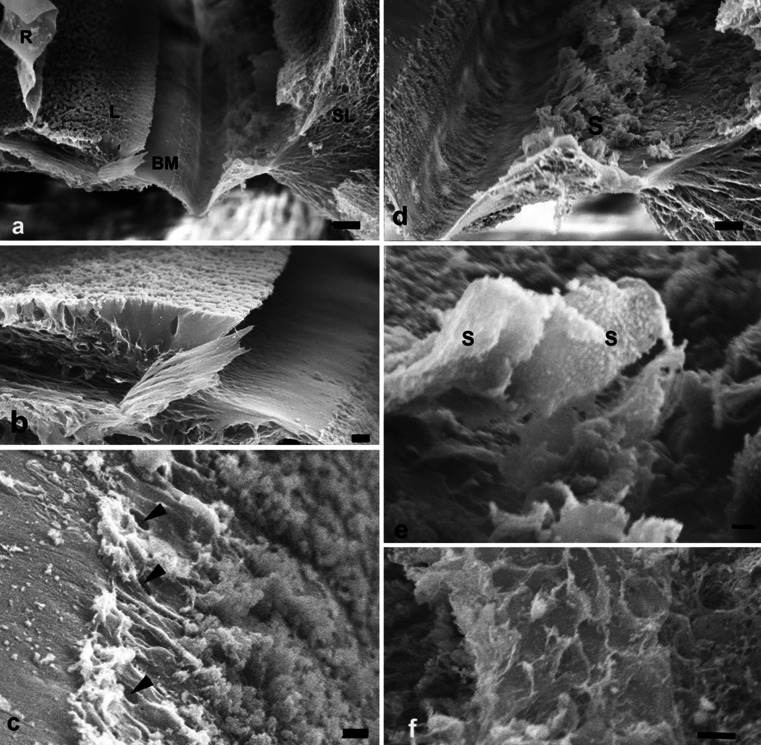
Decellularized human temporal bone. **a** A SEM view of the lower portion of the scala media showing: Reissner’s membrane (*R*), spiral limbus (*L*), basilar membrane (*BM*) and spiral ligament (*SL*). *Bar* = 25 μm. **b** Spiral limbus (*L*) showing plates of ECM that have apical divisions of the plates. The basal portion of the external sulcus cell region is relatively smooth. *Bar*  10 μm. **c** Habenula perforate (*arrows*) are shown with strands and globular material on the surface of the basilar membrane in the organ of Corti region. *Bar* 2.5 μm. **d** Floor of the scala media showing spirally directed septa-like structures (*S*). *Bar* 10 μm. **e** Higher magnification of the septa showing vertical striations and globular material along their surface. *Bar* 2.5 μm. **f** Spiral prominence showing ECM extensions that appear to have surrounded the base of the spiral prominence epithelial cells. *Bar* 10 μm

After decellularization, all the cells were removed and the extracellular matrix (ECM) was preserved (Fig. 10). Examination of the scala media revealed the ECM of Reissner’s membrane, the spiral limbus, the acellular basilar membrane, spirally directed, septa-like structures along the lateral edge of the basilar membrane, the spiral prominence, fibrous tissue of the spiral ligament and the bony tissues of the otic capsule (Fig. 10a). The spiral limbus appeared as plates of ECM that divided into smaller sections at their apex (Fig. 10b). The basal surface of the region, where the internal sulcus cells were, was relatively smooth (Fig. 10b). At the level of the habenula perforate, there were strands and globular materials. The habenula perforate were clearly seen as holes in the basilar membrane (Fig. 10c). More lateral to the habenula and lateral to the organ of Corti, were prominent spirally-directed, septa-like structures that have been previously described in rodent and human decellularized cochleas by sTSLIM (Fig. 10d). These structures appear as periodic sheets of ECM that extend from the basilar membrane into the scala media with an approximate height of 200 μm (Fig. 10e). The sheets exhibit vertical striations and globular particles along their surface (Fig. 10e). The basilar membrane ended at the fibrous region of the basilar crest and the ECM extended up toward a prominent region of the spiral prominence. Septa resembling the shape of the spiral prominence cells extended out of this matrix (Fig. 10f). There was an obvious lack of holes in the ECM of the lateral wall for the external sulcus cell root processes that is so prominent in rodent cochleae. The ECM of the spiral ligament consists of numerous fibrils of ECM. In decellularized material, the basilar membrane appears very thin compared with fixed tissue and its scala tympani surface is not smooth but contains strands of material (Fig. 10a, d).

### Immunohistochemistry of human basilar membrane

**Fig. 11:**
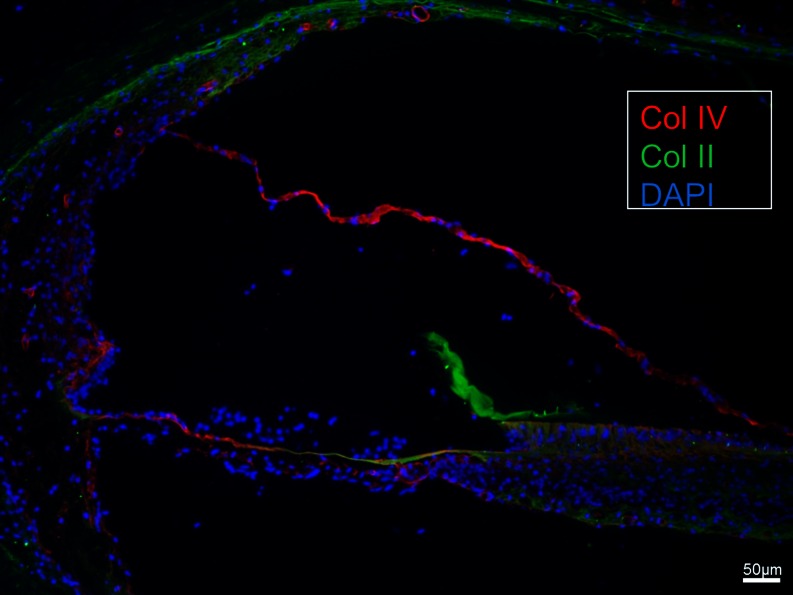
Immunohistochemical expression of collagen II and IV in the human cochlea (II and IV merge). Collagen II is expressed in the BM, spiral lamina, lateral wall and tectorial membrane. Collagen IV is expressed in the basement membranes of the epithelium lining the endolymphatic space, blood vessels and tympanic covering layer. Collagen IV is particularly well expressed at the spiral prominence (*SP*). It also stains the lateral wall of the scala tympani. A modest but clear expression is also seen beneath the stria vascularis cell layers. *DAPI* cell nuclei, apical turn

**Fig. 12:**
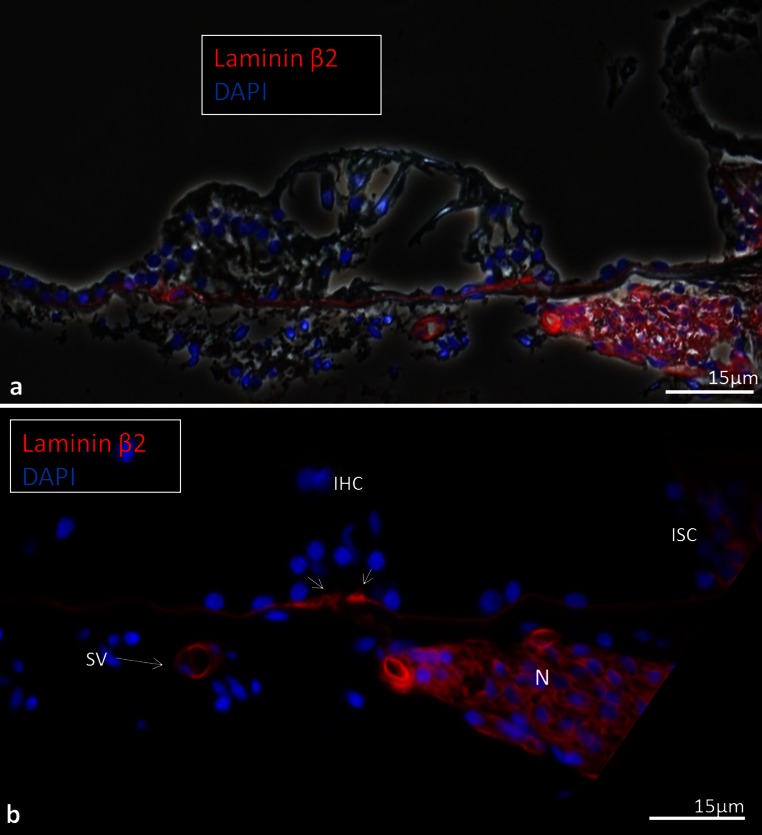
Laminin-β2 immunohistochemistry of the human organ of Corti. **a** Combined bright-field and fluorescent microscopy of the human organ of Corti and lamina spiralis with associated supplying nerves. **b** Fluorescent microscopy of the epithelial basement membrane. The thickness of the basement membrane increases in the habenular region (*small arrows*). *Bar* 20 μm. A spiral vessel shows strong laminin expression. There is no laminin staining in the organ of Corti. Hair and supporting cells are well preserved while the tectorial membrane is distorted. *Bar* 15 μm

**Fig. 13:**
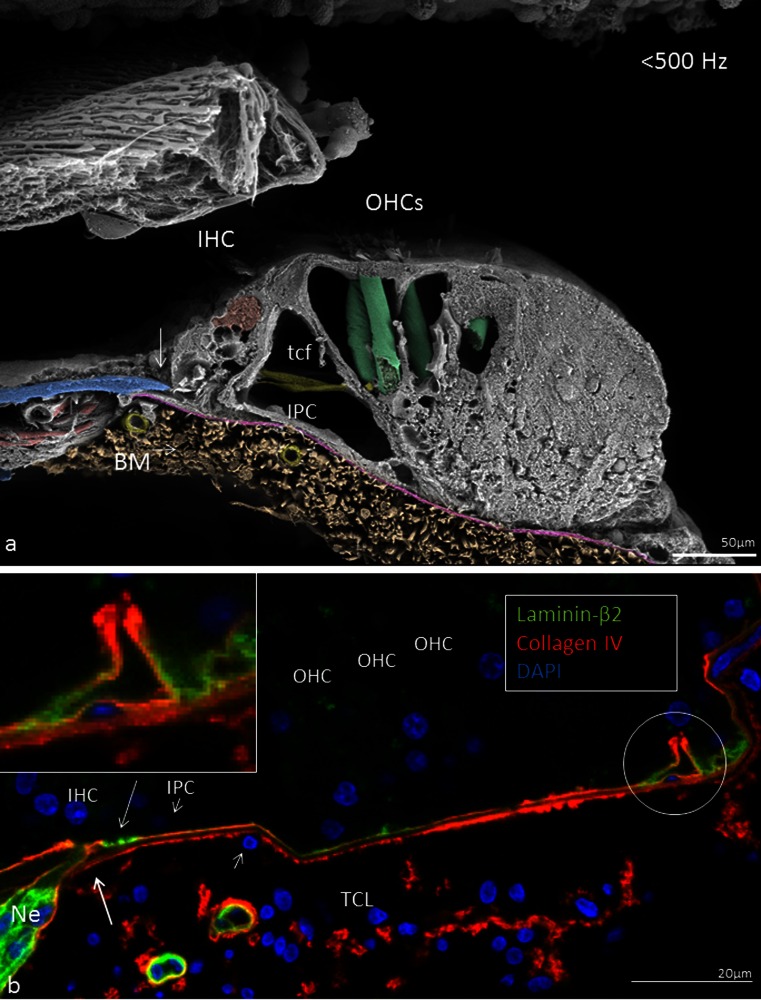
SEM and laminin-β2 and collagen type IV expression in the BM of the human organ of Corti. **a** SEM of the human organ of Corti. The BM is clearly seen as it has separated from the inner pillar foot (fractured) and tympanic lip (*arrow*). *IHC* Inner hair cell, *BM* basilar membrane, *tcf* tunnel crossing fibers, *IPC* inner pillar foot. (×1,000). The figure has been artificially colorized using Photoshop to illustrate the various cell elements. **b** Confocal fluorescent microscopy showing laminin-β2/collagen IV co-expressing BM. The non-stained layer beneath the basement membrane represents the BM proper. A collagen IV immunopositive leaflet is seen below. Tympanic covering layer (*TCL*) expresses collagen IV (<500 Hz). *Inset* shows *encircled area* in higher magnification. Basement membrane expresses laminin-β2 and collagen IV between Claudius cells. *OHC* outer hair cell

**Fig. 14:**
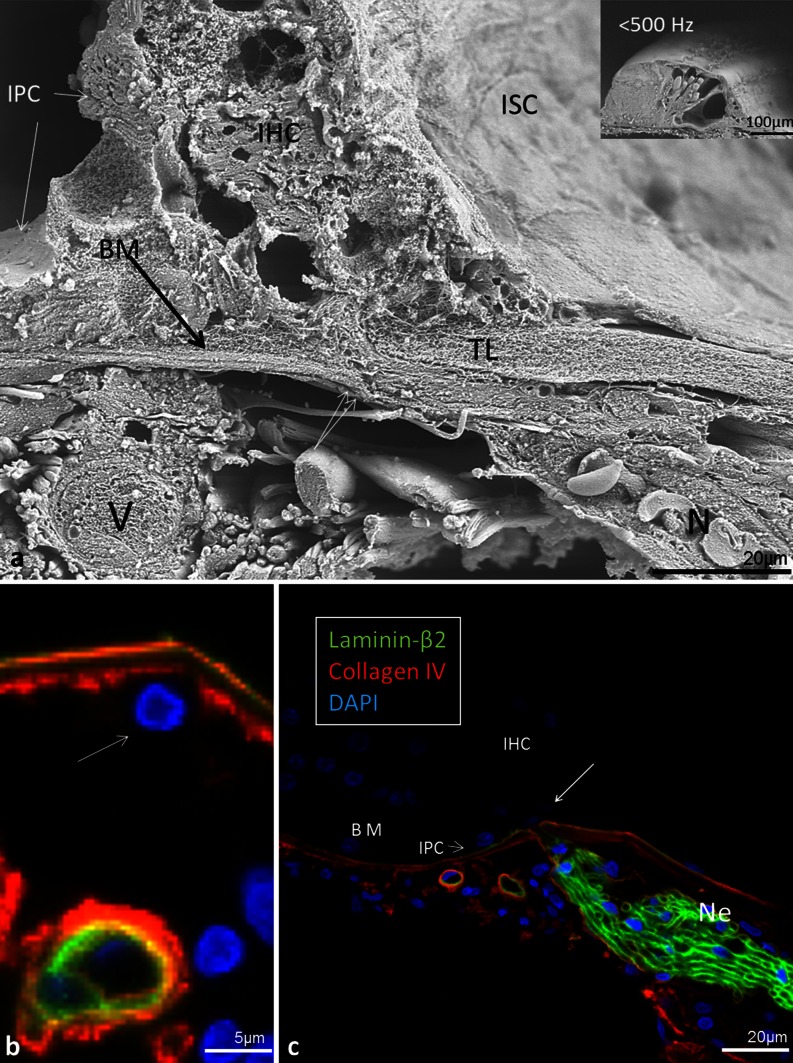
SEM, laminin-β2 and collagen IV expression in the BM of the human organ of Corti. **a** The BM appears interrupted at the habenula (*double arrow*). The tympanic lip (*TL*) forms the medial rim. *Inset* low-power view of the organ of Corti (frequency <500 Hz). **b** Close-up view of vessels shown in Fig. 12b. Laminin/collagen IV staining of the BM. **c** Collagen IV (*red*) stains both the basement membrane of the epithelium and vessel together with laminin and the layer of tympanic covering layer. A concentric ring of collagen IV expression surrounds the spiral vessel. One cell nucleus (*arrow*) adheres to the BM. *IHC* inner hair cell, *BM* basilar membrane, *N* neurons, *IPC* inner pillar cell, *ISC* inner sulcus cells*, V* spiral vessel, *DAPI* nuclear staining. (×1,500). *Bars* (**b**) 15 μm, (**c**) 30 μm

**Fig. 15:**
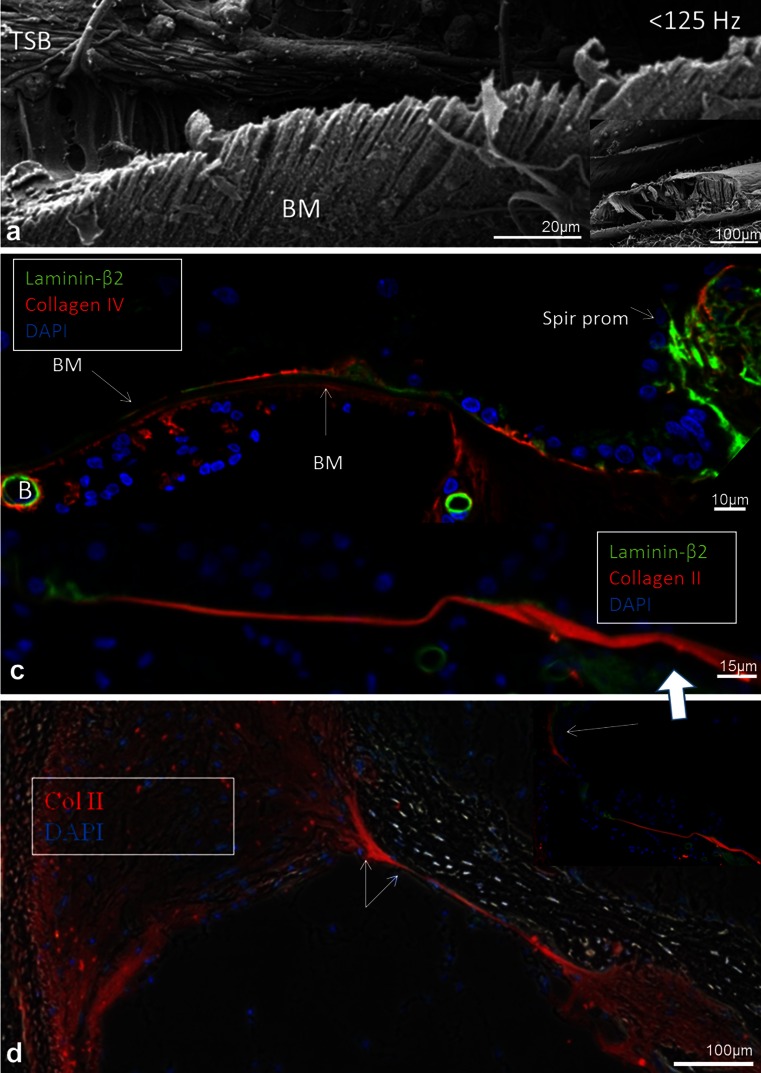
SEM and immunohistochemistry of the human BM. **a** SEM of organ of Corti near the apex (low-frequency region <125Hz) viewed from the scala tympani. BM radial arranged fiber bundles. **b** Laminin and collagen IV expression in the BM. The unstained layer between the two stained layers is collagen II positive. The collagen IV layer continues with the surface wall of the scala tympani while the basement membrane continues beneath the Claudius cells into the spiral prominence where laminin expression is enhanced. **c** BM and OSL stain positive for collagen II. The positively stained BM layer corresponds to the non-stained layer seen in (**b**). At the Claudius cell region collagen II expression is weak while laminin expression is stronger (*left*). **d** In a cochlea from a patient with malignant intracranial tumor the collagen II layer is still preserved but there is no TCL or collagen IV layer present. The BM narrows (*right arrow*) before it expands in the spiral ligament (*left arrow*)

The basement membrane formed a continuous sheet beneath the epithelium margining the endolymphatic space including the organ of Corti. It co-expressed laminin-β2 and collagen IV (Figs. 11, 12, 13, 14, 15). It was thicker at the rim of the habenula perforata and continued as surface lining of the neural openings surrounding the nerve bundle (Fig. 11). The basement membrane continued beneath the inner sulcus cells and surrounded the cell coats of the inter-dental cells of the spiral limbus. Laterally, it persisted under the Claudius, Boettcher (basal turn) and outer sulcus cells. At the spiral prominence, laminin-β2 and collagen IV expression was greatly enhanced (Figs. 11, 15b). At some Hensen/Claudius cells, prominent intra-epithelial projections of laminin-β2/collagen IV expressing basement membranes were seen (Fig. 13). Cell nuclei could sometimes be identified between the BM layers. There was no expression of laminin-β2 or collagen IV within the organ of Corti.

**Fig. 16:**
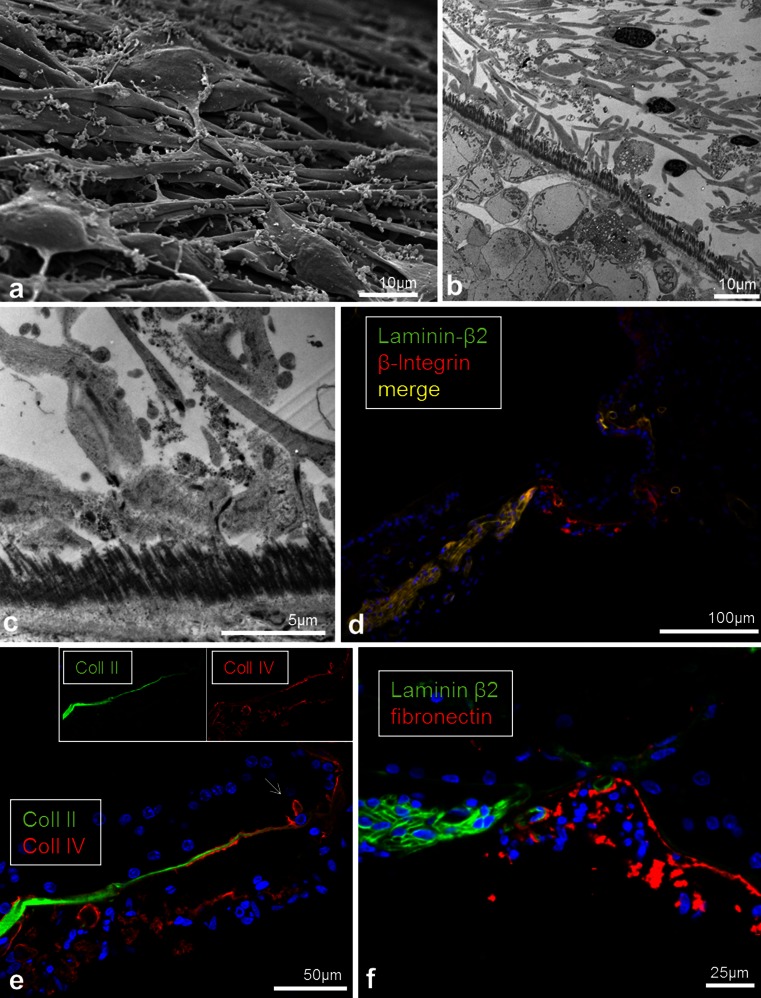
SEM, TEM and immunohistochemistry of the human BM **a** SEM of the TCL at the basal turn. Flat cells have long cytoplasmic processes and small protrusions. **b**, **c** TEM of tangentially sectioned human BM at Hensen’s cells at second turn. Osmium-stained fibrils or “acoustic strings” are observed between the TCL and the basement membrane. **d** Integrin expression in lamina nerve fibers and BM. **e** Collagen II and IV expression in the BM. **f** TCL expression of fibronectin. *Bars* (**d**) 100 μm, (**e**) 50μm, **f** 25 μm

Beneath the basement membrane the BM “proper” expressed collagen II (Figs. 11, 15c, 16e) and collagen XI (not shown). The spiral lamina and tectorial membrane also expressed collagen II. Collagen II expression was often reduced beneath the Claudius’ cells, while lateral to the basilar crest it was prominent (Fig. 15d). Beneath the collagen II layer was a collagen IV stratum (Fig. 13b, 14b, g, 16e). It was associated with the TCL that also expressed collagen IV. At the basilar crest, the TCL tapered the surface wall of the scala tympani. No TCL or collagen IV expression was observed in the cochlea with malignant tumor. The TCL showed strong expression of the glycoprotein fibronectin and the trans-membrane receptor β-integrin.

There was no expression of elastin in the BM. However, a moderate expression of elastin was found in the spiral ligament in the hook region among the type III fibrocytes near the bony wall. There was also some expression of elastin in the tissue margining the BM.

## Discussion

The human ear is exquisitely sensitive to sound. At behavioral thresholds, the BM produces minute vibrations in the range of 0.1 nm, a distance similar to the diameter of a single hydrogen atom (Johnstone et al. 1986; Ruggero et al. 1997; Chen et al. 2011). Frequency resolution and sound sensitivity depend on the physical properties of the BM such as its gradient stiffness, which alters by a factor of 100 from base to apex in human cadaver ears (Békésy 1960; Gummer et al. 1981; Emadi et al. 2004). Due to inner ear fluid damping, the superior sensitivity rests on a 1000-fold, hair cell-based amplification (Kemp 1978; Flock et al. 1986; Rhode 1971). Recent findings suggest that there are probably additional filtering mechanisms in the organ of Corti prior to the excitation of the inner hair cells (Ramamoorthy et al. 2014).

Current progress in electrical stimulation of the cochlea to treat patients with profound and even partial deafness imposes more knowledge about the structure and function of the BM. At present, cochlear implantation (CI) is performed even in patients with residual hearing, with a high level of preservation of cell anatomy and function. Advanced atraumatic techniques at surgery and enhanced electrode designs have been developed. However, the intra-cochlear electrode arrays’ possible interference with the BM is an as yet unresolved problem. The purpose of the present study was to shed more light on these issues from an anatomical/physiological view-point. Surgeons ”on the horns of a dilemma” have to choose between too shallow or deep insertion risking either inadequate functional results with CI or traumatic BM rupture with loss of residual hearing.

We examined size, gradient structure and macromolecular composition of the human BM using optimally fixed normal human cochlear tissue. The organ of Corti displayed a distinctive basement membrane with a lamina lucida and densa. It serves as a border against the other strata of the BM. Previous investigations have shown that it consists of laminin, collagen IV, nidogen and the heparan sulfate proteoglycans (HSPGs) perlecan and agrin (Yurchenco 2011; Timpl and Brown 1996; Tsuprun and Santi 2001). Contrary to findings in man by Ishiyama et al. (2009), we noted distinct expressions of both laminin and collagen IV beneath the inner and outer hair cells. Ishiyama et al. (2009) studied bones from aged people collected at autopsy. Cryosections were analyzed for collagen IVα2, nidogen-1, tenascin-C and laminin-β2. There was only faint nidogen and collagen IVα2 immunoreactivity but positive immunoreaction to laminin-β2 in the BM. No expression was detected underneath the inner and outer hair cells. We found consistent laminin-β2 expression from the basal to the apical portion of the cochlea. The reason for the different results may be that we used polyclonal type IV collagen antibodies while Ishiyama et al. used monoclonal antibodies against collagenIVα2. Another reason could be that we used freshly fixed cochleae. Type IV collagen in mammals is derived from six genetically distinct α-chain polypeptides (α1–α6) (Kalluri 2003) but assemble only in three heterotrimers: α1α1α2, α3α4α5 and α5α5α6 (Khoshnoodi et al. 2008). According to these authors, there is a high degree of conservation of expression of these proteins in the human inner ear compared to rodents.

The present study showed enhanced laminin-β2 and collagen IV expression near the basilar crest, probably owing to a multi-layered arrangement of basement membrane. Concurrently, collagen II staining was reduced, consistent with TEM findings of extreme thinning of the BM fibrous layer beneath the Claudius cells. Remarkably, axons within the organ of Corti showed no surrounding basement membranes with laminin-β2 or collagen IV expression.

The pillar and Deiter cells showed tonofilament anchors near the BM where the basement membrane was structurally modified. The cytoskeleton shaped solid cones of microtubules within a denser cytoplasm associated with the basal plasmalemma. In mice, these cones were found to contain cores of actin surrounded by β-tubulin expressing microtubules (Parsa et al. (2012). As earlier indicated, pillar and Deiter cell feet are not directly attached to the BM (Angelborg and Engstrom 1972) but presumably form junctional complexes whose nature is still unknown (Parsa et al. (2012). These junctions may transfer mechanical vibrations from the BM to the reticular lamina and also from the OHCs/Deiter cells back to the BM.

The BM observed with SEM seemed to correspond to the collagen II layer. It co-expressed collagen II and XI (not shown here). With TEM, this layer appeared less homogeneous. It contained radial fibers believed to act as frequency filters. We named this layer the “BM proper”. It runs from the tympanic lip to the basilar crest. In the chinchilla, the BM consists of radial fibrils with a rectangular shape, 140 A in diameter and different collagens in a matrix (Tsuprun and Santi 1999). In man, various sized fiber bundles were found at different levels in the cochlea. The matrix was not molecularly identified. Tenascin immunostaining gave no consistent results.

IHCs were positioned above the rim of the tympanic lip. They seemed anchored on a fairly rigid fundament suggesting that IHCs are little impacted by direct mechanical stimulation from the BM. Instead, their stereocilia may be subjected to indirect forces. The thinnest part of the BM in the low-frequency region (second and third turns) was beneath the Claudius cells. This seems to be the most vibration-sensitive part and allows the BM to act as a hinge. The design may be consistent with the concept that vibrations of the organ of Corti are driven largely by the OHCs, particularly at low sound levels. However, at the hook, the situation was reversed. A gradient decrease in BM thickness was found from lateral to medial and the thinnest radial width was at the inner pillar cell (approx. 1 μm) while the laterally BM width was almost 5 μm. There was a more than 20 times difference in BM thickness at the hook and second turn at the Claudius cell level. Together, this may suggest that BM motion pattern is virtually different at various locations in the human cochlea. Large thickness variations were also found by Bhatt et al. (2001) who noted a significant age-related thickening of BM, limited to the basal cochlear region. Since our specimens were mostly from individuals below 60 years of age, we do not believe that these dimensions reflect pathology.

Decellularization of the inner ear shows the distribution of the extracellular matrix. Many structures within the scala media were recognizable and the structure of the matrix was clarified due to the removal of the cells. Since the cochlea was not perfused with the decellularization solution, there appeared to be more cell debris left in the cochlea compared with previous observations on the mouse cochlea (Santi and Johnson 2013). The external sulcus cells have a prominent basement membrane and the smooth surface that is observed in that region is likely to be the surface of the basement membrane. The habenula were observed but material (i.e., strands and globules) begin there and extend over the region of organ of Corti cells. Lateral to the organ of Corti and in the Boettcher cells region were numerous septa-like structures. These structures appear to be covered with basement membrane (Fig. 10) and were likely extensions of the ECM between Hensen/Boettcher cells. The basement membrane specialization may correspond to the observations made with immunohistochemistry and TEM. It is unclear why these septa-like structures are present and more numerous and higher in the human compared with rodent cochlea. Their function is unknown. ECM projections were also observed in the spiral prominence epithelial cell region and again are probably extensions of the ECM between these cells. Notable was the lack of the holes for root process in the basement membrane that extended up the lateral wall to the spiral prominence. These holes were very prominent in the mouse cochlea (Santi and Johnson 2013).

The function of the TCL layer is unclear. It contains numerous free cells that may have phagocytic properties (Angelborg and Engström 1974). It may be fundamental for the maintenance of the BM strata. In a specimen of malignant tumor-damaged cochlea with no cell components, the collagen IV layer was absent (Fig. 14d). However, the collagen II layer was unbroken indicating that it is not dependant on an intact TCL. TCL thickness increased apically as the BM widened, except most apically where it seemed to disappear in one specimen analyzed by SEM. Its thickness diverged beneath IHCs and OHCs with an abrupt reduction in thickness below the OHCs after 1.5 turns. According to Bhatt et al. (2001), there is a significant amount of thinning of the TCL with age, with a reduced number of cells at all cochlear locations. At the same time, the fiber thickness of the BM increased significantly with age at the most basal part of the cochlea. These authors found no difference between a control and “indeterminate” presbycusis patient group suggesting that TCL thickness variations may not influence BM stiffness and hearing sensitivity in the low-frequency regions.

Cell processes displayed 2.5-nm microtubules. Microtubules establish cell figure and influence internal cell movements, intracellular organelle transport and locomotion. These cells converge with cells lining the lateral wall of the scala tympani. One may speculate that the cells could form a network that interacts with stretching fibers present in the spiral ligament (Jenkins et al. 1983). Elastin expression seemed to match the anchoring system recently described in the spiral ligament in some mammals. The system includes type III fibrocytes at the bony wall of the cochlea, stretching fibers to the basilar crest, F-actin, myosin and talin (Henson et al. 1985; Kuhn and Vater, 1997; Kelly et al. 2012). Kelly et al. (2012) found contractility in type III cochlear fibrocytes that was dependent on non-muscle myosin II and intercellular gap junction coupling. Cell complexes between the otic capsule and radial attachment sector of the BM could influence tension and micro-mechanics of the BM at the very low base. Further studies are necessary to verify this in man.

The present findings may add to our understanding of the structure and function of the human BM to value potential effects of CI electrode insertions and positioning. CI surgery nowadays permits preservation of residual hearing (so-called electro-acoustic hearing or EAS). Short electrodes can stimulate neurons in the high-frequency region while residual low-frequency hearing is maintained. Patients may receive both acoustic and electric hearing. However, it is not uncommon that low-frequency hearing deteriorates from implantation (Gantz et al. 2009; Adunka et al. 2013; Erixon et al. 2012). The hearing loss can be both conductive and sensorineural and may impinge on frequencies tonotopically not linked to the electrode position. Hearing loss can be reasoned by a physical interference with the miniscule vibrations and the BM travelling wave influencing its dynamic properties at a level more inferior in the cochlea. The jeopardy enhances with deeper electrode insertion since the width of the BM increases apical wards. Concomitantly, the scala tympani width decreases. The BM occupies only 10 % of the scala tympani width at the base but 80 % in the apex. Therefore, the risk for the electrode to facade the BM apically is higher. Consequently, both fluid displacement and electrodes’ physical interference may compromise BM motions following deep insertion.

From the following, it is irrefragable that cochlear physiology and hearing, even with today’s miniaturized electrodes, cannot be impervious to their introduction in the cochlea. The dimensions of the BM change longitudinally and radially with increased fragility apical wards. After one turn, the BM thickness decreased almost 50 % beneath the OHCs, being mostly less than 1 μm. There is an extreme thinning of the BM under the Claudius cells in the second turn. Even though TCL thickness increases, it probably offers little resistance to an electrode that can easily traumatize or perforate the BM. Another factor is the fluid volume of the scala tympani that is occupied by the electrode. Displacement of fluid may alter the BM vibration properties since both are considered as a “mass-spring” system influencing resonant properties. This may explain some loss of residual hearing experienced after electrode insertion in partially deaf patients.

In a recent study, we found that hearing loss in some patients progressed over time (Erixon and Rask-Andersen, in manuscript). Similar findings have been reported by others (Gstoettner et al. 2006; Luetje et al. 2007). An explanation for this may be a foreign body reaction around the electrode. The TCL may generate protective responses due to near-by located infection-prone areas. Spiral vessels may recruit leucocytes into the cochlear tissue. This pathway could be triggered in CI surgery leading to inflammation and fibrosis around the electrode array. Cells and fibrous elements in the TCL were found to express collagen IV and also fibronectin and the trans-membrane receptor β-integrin. Fibronectin fragments have been shown to be chemotactic for human peripheral blood monocytes and EDA-fragments (Extra Domain A). These can promote expression of genes involved in the inflammatory response and activate the toll-like receptor (TLR4), the signaling receptor stimulated by bacterial lipopolysaccharide (Okamura et al. 2001). One way to avoid these reactions may be to use anti-inflammatory drugs.

In conclusion, there are several important similarities and differences between the morphology of the BM in humans and animals that are as follows. The primary structure of the BM is the same. It is a primarily extracellular matrix that supports the organ of Corti and other cells and extends between the osseous spiral lamina and the basilar crest. Its width increases and thickness decreases as it travels apically. It contains a continuous basement membrane on its scala media side and blood vessels and mesothelial cells on its scala tympani side. It contains a bundle of radial filaments that are rich in collagen type II. It does not appear to contain elastin. It differs from animals in that it does not appear to contain a distinct pars tecta (arcuate) and pectinata. It appears more cellularized and vascularized compared with animals. It contains a single rather than two bundles of radial filaments. It does not appear to contain a region of homogenous ground substance in association with the radial filament bundle. It contains a large, cellularized tympanic layer. It appears to contain type IV collagen associated with the radial bundles and the septa-like structures on the apical (i.e., scala media) side that are larger and more developed compared with animals. It is not known whether these morphological differences also represent functional differences between the human and animal basilar membrane.

## Conclusions

Laser confocal microscopy, high resolution scanning (SEM) and transmission electron microscopy (TEM) showed that the human BM thickness varies radially and longitudinally (mean 0.55–1.16 μm). BM is thinnest and probably most vibration-sensitive at the outer pillar feet/Deiter cells at the OHCs and laterally. The BM consists in four separate layers: (1) epithelial basement membrane positive for laminin-β2 and collagen IV, (2) BM “proper” composed of radial fibers expressing collagen II and XI, (3) layer of collagen IV and (4) tympanic covering layer (TCL) expressing collagen IV, fibronectin and integrin. Unlike in animals, it does not contain a distinct pars tecta (arcuate) and pectinata. The inner pillar and IHCs seem to be situated on a fairly rigid part of the BM. The gradient design of the BM suggests that its vulnerability increases apical wards when performing CI surgery.

## Electronic supplementary material

Below is the link to the electronic supplementary material.

ESM 1 (PDF 235 kb)
