# Supplementary material for: Macromolecular organization and fine structure of the human basilar membrane - RELEVANCE for cochlear implantation
Source: Cell Tissue Res. 2015 Feb 7;360(2):245–62. doi: 10.1007/s00441-014-2098-z (PMC4412841; doi:10.1007/s00441-014-2098-z)
Supplement: Supplementary file 1 — (PDF 235 kb) [file 441_2014_2098_MOESM1_ESM.pdf]

- Unlike animals human BM does not contain a distinct pars tecta (arcuate) and pectinata.
- BM thickness varies both radially and longitudinally. The minimum thickness of the BM (proper) fibrous layer at the Claudius cells differed more than 20 times (0.2 - 4.8 microns) between high and low frequency regions (hook and second turn).
- BM width increases and thickness decreases apically in the cochlea.
- Human BM is thinnest and probably most vibration-sensitive at the outer pillar feet/Deiter cells at the OHCs and laterally.
- BM and extra-cellular protrusions occur between cells in the organ of Corti in man.
- The inner pillar and inner hair cells are situated on a fairly rigid part of the BM.
- BM is more fragile apically when performing cochlear implant surgery.

The human BM consists of four layers

1) Epithelial basement membrane positive for laminin-β2 and collagen IV
2) BM “proper” composed of radial fibers expressing collagen II, and XI
3) Layer of collagen IV
4) Tympanic covering layer (TCL) expressing collagen IV, fibronectin and integrin

- Cochlear implant surgery nowadays permits preservation of residual hearing (so-called electro-acoustic hearing or EAS).
- A progressive low-frequency hearing loss is often noted after EAS surgery. Its etiology is unknown.
- It may be caused by a foreign body reaction triggered by the TCL leading to inflammation and fibrosis around the electrode array.
- TCL was found to express fibronectin and the trans-membrane receptor β-integrin. It may promote expression of genes activating the toll-like receptor (TLR4) causing inflammation.
- To avoid these reactions, use of anti-inflammatory drugs before and during surgery may be beneficial.
